# Supplementary material for: Prevalence of antimicrobial resistance in Tanzania: A systematic review and meta-analysis
Source: PLoS One. 2026 Apr 21;21(4):e0346433. doi: 10.1371/journal.pone.0346433 (PMC13098951; doi:10.1371/journal.pone.0346433)
Supplement: Attachment — Submitted filename: ESKAPE-E Manuscript Review.pdf [file pone.0346433.s004.pdf]

Review: Prevalence of Antimicrobial Resistance in Tanzania: A Systematic Review and Meta Analysis--Manuscript Draft

Introduction:

Key words listed: Antimicrobial resistance, ESKAPE-E, AWaRe, Tanzania

The review concisely explains the term antimicrobial resistance (AMR), particularly pertaining towards the ESKAP-E microbes, and the human error that increases the prominence of drug resistance without mentioning the method of action as to how the changes in drug efficacy manifests. When discussing Tanzania, there is a thorough explanation as to why that particular demographic was selected to examine the urgency of rising antimicrobial resistance within in the Methods section. The acronym AWaRe, is listed during the discussion but significance is not detailed.

The introduction concludes with the study's purpose, which is to conduct a systematic review and meta-analysis to determine the overall prevalence of antibiotic resistance in Tanzania, but the way it is written makes the investigation's objective somewhat unclear due to being split across several sentences and presenting itself as background information rather than a specific objective. What can be inferred is that there is a gap in consolidated national data on antibiotic resistance to ESKAP-E microbes in Tanzania, and the systematic review and meta-analysis was conducted to fill this gap. And the aim is to provide a comprehensive overview of resistance prevalence across pathogens and antibiotics with the intention to reform national policy and AMR strategies.

Methods:

The selection begins with the registration number of the review's protocol with PROSPERO, rather than a description of protocol and how it's foundation aligned with the study objective. Additionally, the subsection, 'S*ettings*' is better fit for the introduction, rather than its current placement in the methodology; in order to provide more robust evidence as to why Tanzania was the selected site for investigation, prior to stating the study's objective.

When discussing procedures, the author stated that they adhered to the Preferred Reporting Items for Systematic Reviews and Meta-analysis (PRISMA) guidelines for reviewing, analyzing, and reporting systematic reviews but do not explain specifically what that procedure entails. The terms listed for inclusion and exclusion literature were effectively depicted, although the number of outcomes for each search was omitted, with

subsequent searches not represented in a way that expresses how the literature was funneled down to the most relevant information in order to shape the study. It is stated that upon following their 4 steps for selection of inclusion data, that they were able to select 28 articles from a pool of 18,265.

Animal studies were listed in the exclusion criteria, and due to the nature of systemic reviews and meta-analysis, it is unlikely that there would be ethical conflicts during the data collection. Moreover, the author states that they were not applicable to ethical considerations. This study is repeatable.

Data Analysis:

Due to the study's presumed objective to combine resistance proportions from multiple individual studies to generate an overall prevalence estimate, the selected meta-analysis with the utilization of forest plots and the I^2 statistic for the examination of heterogeneity among the selected studies, was the most effective tool. The data and charts are relevant to the study and depict resistance outcomes when measured with the antibiotics .

Table 1 is entitled "Study population, sampling methods, sample size, number of isolates and sample sources of the selected studies", but the data depicted lists as the author, study population, sample size, and number of isolates. This can be more aptly titled, data source, study demographic, sample size, and isolates. Additionally, there are discrepancies that disrupt the presumed validity between sample size and the number of isolations from the samples; specially articles from Manyahi et al., 2014, Seidman et al., 2016, Mikomangwa et al., 2020, Masoud et al., 2022, which are depicted in the table as having sample sizes smaller than their isolates. No abbreviations were used, and footnotes were not applicable.

Results:

The analysis listed a thorough depiction of key findings between 17 antibiotics and all ESKAPE-E, with the addition of several microbes not highlighted in the investigation but had data and subsequent outcomes that the investigator found important to note. The author states that study limitations consisted of the lack of generalizability of the findings to the broader population, due to the majority of the studies were conducted in hospital or rural settings. However, the author does not include the fact that 46% of their chosen sample demographic were children under 5 as a limitation. This factor also affects the study's generalizability and external validity through the possibly of selection bias.

This study was conducted throughout the years 2014-2024. The manuscript reads that 17.9% of the studies used were published in 2020 alone; while 14.3% were published in 2016, 2019, and 2022, this factor does not appear to be included in the limitations. This potentially impacts the validity due to unique environmental factors that could have varied throughout that 10-year span which can produce a sample bias. Subgroup analyses were conducted to investigate potential drug resistance to specific pathogens associated with each antibiotic but there were not conducted to explore the data collection period as a potential factor influencing their results. The manuscript states that they used the Joanna Briggs Institute's Quality Assessment Tool for Cross-Sectional Studies, with an included table, but the assessment tool was not explained. Furthermore, the author used other study designs such as longitudinal, prospective and retrospective cohorts that cannot be used with their selected tool to account for bias.

Discussion:

This section offers a summary of the findings as well as lists the proportion of resistance per antibiotic examined in association with ESKAPE-E pathogens.

Conclusion:

The purpose of the study is sufficiently restated without further mention of the analysis results, and author provide clear recommendations for policy, practice, and future research.
