# Supplementary material for: Prevalence of antimicrobial resistance in Tanzania: A systematic review and meta-analysis
Source: PLoS One. 2026 Apr 21;21(4):e0346433. doi: 10.1371/journal.pone.0346433 (PMC13098951; doi:10.1371/journal.pone.0346433)
Supplement: Attachment — Submitted filename: Response to reviewers_PLOS one.pdf [file pone.0346433.s006.pdf]

Review of manuscript- PLOS One<br>**Tittle: Prevalence of Antimicrobial Resistance in Tanzania: A Systematic Review and Meta-Analysis**	
Dear Editor,<br><br>The authors thank the editor for reviewing our work.<br><br>Please see our point-by-point response to the reviewers' comments below and find our revised manuscript attached.<br><br>On behalf of the co-authors,<br>Kind regards,<br><br>Charles Basil Kafaiya<br>*kafaiyacharles@gmail.com*<br>*Research Scientist,*<br>*National Institute for Medical Research, Dar es salaam, Tanzania*	
**Reviewer 1**	**Response**
**General and abstract section**<br>Authors aimed to perform the systematic review and meta-analysis to present prevalence of AMR in Tanzania. Authors may clarify if they included animal and environmental AMR studies and if not why they are excluded (just to confirm)	We thank the reviewer for this important observation. In this systematic review and meta-analysis, we focused exclusively on studies reporting antimicrobial resistance (AMR) in humans in Tanzania. Animal and environmental AMR studies were not included. The primary rationale for this exclusion is that the scope of our review was designed to inform clinical and public health interventions directly related to human health. Including animal and environmental studies would have broadened the scope substantially and introduced additional heterogeneity, potentially limiting the interpretability of pooled prevalence estimates
**Results section**<br>Authors included ESKAPE-E bacterial organisms of which majority are multidrug resistant. All except Streptococcus spp. are intrinsically resistant to penicillin/ampicillin. These organisms are mostly acquired in healthcare settings and the most of access antibiotics from AWaRe classes are rather ineffective. They may apply to community infections.<br>KP as intrinsically resistant to penicillin/ampicillin should be removed from analysis and the same for A. baumannii and other from the group. Klebsiella pneumoniae exhibits intrinsic resistance to ampicillin due to the presence of the SHV-1 penicillinase gene on its chromosome. The antibiotic resistome concept is predicated on the understanding that soil-dwelling environmental bacteria are producers of antimicrobial compounds. Thus, many components of the resistome must have evolved long before the use of antibiotics became clinically common. Based on knowledge about the bacteria in the group one may suggest to reanalyze data and focus on	We acknowledge the observation regarding intrinsic resistance in *Klebsiella pneumoniae*, *Acinetobacter baumannii*, and other ESKAPE-E organisms. In our systematic review, we reported findings exactly as presented in the previous studies to provide a comprehensive overview of antimicrobial resistance patterns in Tanzania. Excluding organisms or antibiotics with intrinsic resistance may introduce bias by underestimating the prevalence of multidrug resistance reported in the literature.<br>Furthermore, the review employed the stated inclusion criteria consistently, focusing on the resistance data reported in the selected human studies

watch and reserve antibiotics and to categorize according settings	
**Reviewer 2**	**Response**
**Introduction**<br><br>Key words listed: Antimicrobial resistance, ESKAPE-E, AWaRe, Tanzania<br><br>The review concisely explains the term antimicrobial resistance (AMR), particularly pertaining towards the ESKAP-E microbes, and the human error that increases the prominence of drug resistance without mentioning the method of action as to how the changes in drug efficacy manifests. When discussing Tanzania, there is a thorough explanation as to why that particular demographic was selected to examine the urgency of rising antimicrobial resistance within in the Methods section. The acronym AWaRe, is listed during the discussion but significance is not detailed<br><br>The introduction concludes with the study's purpose, which is to conduct a systematic review and meta-analysis to determine the overall prevalence of antibiotic resistance in Tanzania, but the way it is written makes the investigation's objective somewhat unclear due to being split across several sentences and presenting itself as background information rather than a specific objective. What can be inferred is that there is a gap in consolidated national data on antibiotic resistance to ESKAP-E microbes in Tanzania, and the systematic review and meta-analysis was conducted to fill this gap. And the aim is to provide a comprehensive overview of resistance prevalence across pathogens and antibiotics with the intention to reform national policy and AMR strategies	Thank you for the insightful and constructive comments. We have carefully revised the manuscript to address the concerns raised
**Methods**<br><br>The selection begins with the registration number of the review's protocol with PROSPERO, rather than a description of protocol and how its foundation aligned with the study objective. Additionally, the subsection, 'S*ettings*' is better fit for the introduction, rather than its current placement in the methodology; in order to provide more robust evidence as to why Tanzania was the selected site for investigation, prior to stating the study's objective<br>When discussing procedures, the author stated that they adhered to the Preferred Reporting Items for Systematic Reviews and Meta-analysis (PRISMA) guidelines for reviewing, analyzing, and reporting systematic reviews but do not explain specifically what that procedure entails. The terms listed for inclusion and exclusion literature were effectively depicted, although the number of outcomes for each search was omitted, with subsequent searches not represented in a way that expresses how the literature was funneled down to the most relevant information in order to shape the study. It is stated that upon following their 4 steps for selection of inclusion data, that they were able to select 28 articles from a pool of 18,265	Thank you for the highlighted observations, the authors have revised the method section

**Data Analysis**<br><br>Due to the study's presumed objective to combine resistance proportions from multiple individual studies to generate an overall prevalence estimate, the selected meta-analysis with the utilization of forest plots and the I^2 statistic for the examination of heterogeneity among the selected studies, was the most effective tool. The data and charts are relevant to the study and depict resistance outcomes when measured with the antibiotics<br><br>Table 1 is entitled "Study population, sampling methods, sample size, number of isolates and sample sources of the selected studies", but the data depicted lists as the author, study population, sample size, and number of isolates. This can be more aptly titled, data source, study demographic, sample size, and isolates. Additionally, there are discrepancies that disrupt the presumed validity between sample size and the number of isolations from the samples; specially articles from Manyahi et al., 2014, Seidman et al., 2016, Mikomangwa et al., 2020, Masoud et al., 2022, which are depicted in the table as having sample sizes smaller than their isolates. No abbreviations were used, and footnotes were not applicable	Thank you for this insightful observation. We acknowledge that the original title of Table 1 did not accurately reflect all the data elements presented. The table title has now been revised to "Authors, study population, sample size, and number of isolates from the selected studies" to better align with the information displayed<br><br>Regarding the observed discrepancies between sample size and the number of isolates, we would like to clarify that in several of the included studies, multiple isolates were obtained from a single participant or sample source. This occurs when more than one bacterial species or strain is cultured from the same individual specimen, resulting in the number of isolates exceeding the number of participants or samples
**Results**<br><br>The analysis listed a thorough depiction of key findings between 17 antibiotics and all ESKAPE-E, with the addition of several microbes not highlighted in the investigation but had data and subsequent outcomes that the investigator found important to note. The author states that study limitations consisted of the lack of generalizability of the findings to the broader population, due to the majority of the studies were conducted in hospital or rural settings. However, the author does not include the fact that 46% of their chosen sample demographic were children under 5 as a limitation. This factor also affects the study's generalizability and external validity through the possibly of selection bias<br><br>This study was conducted throughout the years 2014-2024. The manuscript reads that 17.9% of the studies used were published in 2020 alone; while 14.3% were published in 2016, 2019, and 2022, this factor does not appear to be included in the limitations. This potentially impacts the validity due to unique environmental factors that could have varied throughout that 10-year span which can produce a sample bias. Subgroup analyses were conducted to investigate potential drug resistance to specific pathogens associated with each antibiotic but there were not conducted to explore the data collection period as a potential factor influencing their results. The manuscript states that they used the Joanna Briggs Institute's Quality Assessment Tool for Cross-Sectional Studies, with an included table, but the assessment tool was not explained. Furthermore, the author used other study designs such as longitudinal, prospective and	Thank you for the highlighted observations, the authors have revised the Result and limitation section

retrospective cohorts that cannot be used with their selected tool to account for bias	
**Discussion**<br>This section offers a summary of the findings as well as lists the proportion of resistance per antibiotic examined in association with ESKAPE-E pathogens	Thanks for the compliment
**Conclusion**<br><br>The purpose of the study is sufficiently restated without further mention of the analysis results, and author provide clear recommendations for policy, practice, and future research	Thanks for the compliment
**Reviewer 3**	
**Abstract**<br>1. Please include justification for conducting the study in the abstract<br>2. Adjust the statement "Heterogeneity was assessed using forest plots". A forest plot is not used to assess heterogeneity. Heterogeneity is assessed using I^2 statistic.<br>3. Is the prevalence of antimicrobial resistance defined by descriptive or meta-analysis?<br>4. Adjust the grammar in this statement: "Resistance to meropenem was lowest among Escherichia coli (0.04 [0.01–0.10]) and Klebsiella spp. (0.07 [0.03–0.15]), with an overall pooled 37 resistance to the ESKAPE-E pathogen of (0.11[0.06–0.19])."<br>5. Specify who this recommendation is targeted at "The variability in resistance patterns underscore the need to re-evaluate empirical treatment protocols (STG/NEMLIT) to ensure effective treatment regimens, strengthening antimicrobial stewardship, enhancing surveillance systems, and promote rational antibiotic use."	1. Thank you for your observation we have included the justification statement for conducting the study in Line 20-23<br>2. Thank you for your observation we have adjusted the statement to indicate the Heterogeneity was assessed using I^2 statistic<br>3. Thanks for the observation, no we have revised the statement to "the prevalence of antimicrobial resistance was estimated through Meta-analysis"<br>4. Thanks for the observation, we have adjusted the statement to look grammatically fine<br>**5.** Thanks for the observation, we have revised the statement and clearly specify that this recommendation is targeted at the Ministry of Health
**Introduction**<br><br>6. Line 47 – 59: Can you quantify the burden of antimicrobial resistance globally and in Tanzania, respectively?<br>7. Clarify the language in this statement: "This phenomenon is particularly noticeable in low- and middle-income countries, where antibiotic abuse is pervasive, infectious disease burdens are high, yet scarce healthcare resources"<br>8. Provide references for the statement: "A variety of factors contribute to the rising rates of Antimicrobial Resistance (AMR) in the country, including over-prescription of antibiotics, self-medication, and inadequate regulatory frameworks for antibiotic use."<br>9. What do you mean by "urgency" in line 68?<br>10. Line 69 -70: describe and cite what other reviews have done or did not do. Also, cite some individual studies on antimicrobial resistance in Tanzania that you referred to in this section.<br>11. Address the grammar in this statement: While several studies have reported resistance patterns for specific	6. Thank you for the observation, we have added statements to show and quantify the burden of AMR globally and in Tanzania<br>7. Thank you for the comment, we have revised the sentence to improve clarity and precision using neutral and academically appropriate language<br>8. Thank you for the comment, we have now provided appropriate references to support this statement<br><br>9. Thank you for the comment, "urgency" refers to the critical public health concern posed by resistant pathogen, we have also revised the sentence to make it clearer, "Despite the critical threat posed by resistant

pathogens, therefore a consolidated analysis that captures the overall prevalence across the country was needed	pathogens, systematic reviews focusing specifically on Tanzania's antibiotic resistance landscape are limited"<br>10. Thank you for the comment, we have revised the section and cited accordingly to support the statements<br>11. Thank you for the comment, we have revised the statement, to improve grammar and clarity
**Method**<br><br>12. I think the information on lines 91 – 98 should be part of your introduction<br>13. State what approach was used to define the research question. If you used the PICO approach, include it in your "Review procedures section."<br>14. Justify why you focused only on studies across 10 years<br>15. In your selection criteria, is there a difference between "studies reporting on the prevalence of antibiotic resistance in bacterial pathogens" and "studies focused on antibiotic resistance"<br>16. Did you predefine the bacterial pathogens and antibiotics analyzed in this review? If yes, you should justify why they were selected<br>17. State how many reviewers conducted the quality assessment?<br>18. Include the statistical package you used to conduct descriptive statistics.<br>19. Usually, only the I^2 statistic is used to evaluate heterogeneity and not the forest plot.<br>20. What cut-off was used to determine the higher value for the I^2 statistic?	12. Thank you for the observation, we have incorporated the statement to the introduction section as advised<br>13. Thank you for the comment, The research question was defined using the PICOS framework, which has now been clearly included in the Review Procedures section as advised<br><br>14. Thank you for the comment, we focused on studies from the last 10 years to align our review with the period most relevant to current national AMR policy and action. Tanzania's National Action Plan on Antimicrobial Resistance 2023–2028 builds on progress and gaps identified during the previous 2017–2022 plan, emphasizing strengthened surveillance, research, and evidence generation to inform policy and practice moving forward. By reviewing literature from the past decade, our analysis captures trends that directly inform and contextualize the implementation, monitoring, and strategic priorities articulated in the current NAP-AMR (2023–2028)<br><br>15. Thank you for the observation, there is no difference between *"studies reporting on the prevalence of antibiotic resistance in bacterial pathogens" and "studies focused on antibiotic resistance";* both refer to studies that report antimicrobial resistance prevalence. We have revised the

	text to remove repetition and avoid any ambiguity<br><br>16. Thank you for the observation, **no** bacterial pathogens or antibiotics were predefined in this review. All studies reporting antimicrobial resistance in any bacterial pathogens were eligible, allowing for a comprehensive assessment of resistance patterns in Tanzania<br><br>17. Thank you for the observation, The quality assessment of included studies was conducted independently by **two reviewers**, with any disagreements resolved through discussion or consultation with a third reviewer to ensure consistency and reliability<br><br>18. Thank you for your observation, we have in cooperated the suggested statement, stating clearly which package was used for descriptive statistics<br><br>19. Thank you for your observation we have adjusted the statement to indicate the Heterogeneity was assessed using I^2 statistic<br><br>20. Thank you for your comment. A cut-off of 75% was used to define the higher value for the I^2 statistic, indicating high heterogeneity among the studies
Results<br>21. More specific numbers should be used instead of percentages to report on the number of studies. When using percentages, maintain consistency in reporting. Also, ensure that the papers conducted at each location are cited.<br>22. Line 186 – 190. You mentioned 17 antibiotics but only listed 10. You should include the other seven. You should define the criteria that were used to categorize resistance as high or low.	21. Thank you for this comment, we have revised the manuscript to report the number of studies alongside percentages, ensuring consistency in percentage reporting throughout the text. In addition, we have cited all studies conducted in each respective location accordingly<br>22. Thank you for this observation, we have revised the statement to include all 17 antibiotics. In addition, we have clarified the criteria used to categorize resistance levels, defining high

	resistance as a prevalence of ≥50% and low resistance as <50%, and this information has now been explicitly stated in the Methods section
**Discussion**<br>23. This is new; it was not mentioned in the result. Ensure that you discuss only the results presented.<br>24. I suggest you focus on interpreting the results rather than repeating them in the discussion section.<br>25. Line 306: "Reserved group." This was not mentioned in the result. You should not bring up new terms or information in your discussion<br>26. You should also focus on connecting the findings of the study to the study objectives. The objectives seem lost somewhere in the discussion<br>27. You should provide explanations and reasons behind each finding from the study<br>28. "These findings are similar to the studies conducted in Iran, USA, India, and Africa (80 83), where higher resistance rates were reported, highlighting the potential regional differences in antibiotic use and resistance patterns." Please, provide more information on these studies.<br>29. You may also want to add some recommendations to the discussion	23. Thank you for the observation, the authors have revised the paragraph<br>24. Thank you for the suggestion, the discussion has been revised to focus more on interpretation and implications of the findings, with reduced repetition of results<br><br>25. Thank you for this observation, the "Reserve group" has now been incorporated into the results section to ensure consistency and to avoid introducing new information in the discussion<br><br>26. Thank you for this suggestion. The discussion has been revised to clearly link the study findings to the study objectives, ensuring better alignment and clarity<br><br>27. Thank you for this valuable comment. The discussion has been revised to include explanations and possible reasons underlying the key findings, supported by relevant literature<br><br>28. Thank you for the observation, we have revised the paragraph, to fit with regional comparison<br><br>29. Thank you for the comment, the discussion has been revised accordingly
**Conclusion**<br>30. "Last resort" is this referring to the reserved group mentioned in the discussion section? If yes, ensure consistency in the terms used across the manuscript	30. Thank you for this comment. Yes, "last resort" refers to the Reserve group. The terminology has been standardized across the manuscript to ensure consistency
**Reviewer 4**	
**1. Introduction**<br>Strengths<br><br>Gives a clear general background on antimicrobial resistance and global importance.	We thank the reviewer for the insightful comments and observations. The authors have carefully addressed all the raised concerns by revising the Introduction to strengthen the literature review, clearly articulate the knowledge gap, and improve

Cites authoritative sources including WHO, NFID, and global AMR estimates.<br><br>Identifies ESKAPE pathogens as critical threats and connects this to clinical relevance.<br><br>Areas for Improvement<br><br>a. Literature Review Depth<br>The introduction describes global and Tanzanian context but does not sufficiently review past systematic reviews or local prevalence meta-analyses. For example:<br><br>EMR surveillance reports (Camara 2023, Silago 2024) are mentioned later but not integrated into the introduction.<br><br>The review does not discuss earlier regional AMR systematic reviews beyond one sentence.<br><br>A stronger introduction should synthesize:<br><br>Regional AMR trends (East Africa 2005–2020)<br><br>Tanzania's National AMR Action Plan (2017–2022)<br><br>Gaps in surveillance and empirical treatment guidelines (STG/NEMLIT)<br><br>b. Clear identification of the knowledge gap<br>The authors state that "comprehensive data remain sparse" but should articulate:<br><br>What has not been synthesized previously<br><br>Why a national pooled prevalence is needed now<br><br>How this research will directly inform Tanzanian AMR policy<br><br>c. Objective Placement and Clarity<br>The last paragraph provides an objective ("to present a thorough overview…") but this should be formatted as a clear study aim:<br><br>"This systematic review and meta-analysis aimed to estimate pooled prevalence of antibiotic resistance among clinically relevant bacterial pathogens in Tanzania from 2014–2024 and evaluate resistance patterns across antibiotic classes using subgroup analyses."	the clarity and placement of the study objective. These revisions have enhanced the contextual depth, policy relevance, and overall clarity of the manuscript
**2. Methods**<br>Strengths<br><br>Protocol registered on PROSPERO (major strength).<br><br>PRISMA guidelines followed appropriately.	We thank the reviewer for the constructive comments on the Methods section. The authors have revised the manuscript to clarify the search strategy, duplicate handling, data extraction procedures, risk of bias assessment, meta-analysis methods, and ethical considerations, thereby improving

Search terms and Boolean operators provided.<br><br>Clear inclusion and exclusion criteria.<br><br>Data extraction and quality assessment tools described.<br><br>Meta-analysis approach with random-effects model is appropriate given expected heterogeneity.<br><br>Areas for Improvement<br><br>a. Search Strategy<br><br>Google Scholar yielded 18,000 results. The authors should specify:<br><br>how many pages were screened<br><br>which filters were used<br><br>what strategies were used to avoid missing key grey-literature but also avoid irrelevant hits.<br><br>b. Duplicates<br><br>Only 416 duplicates removed out of 18,265 – this appears unusually low. Most systematic reviews typically remove 10–30% duplicates. Authors should clarify if:<br><br>deduplication was automated,<br><br>inclusion was restricted by year,<br><br>Google Scholar citations were sorted.<br><br>c. Data Extraction<br>A brief description is provided, but reproducibility requires more detail:<br><br>Was a data extraction form piloted?<br><br>How were discrepancies resolved between the two independent reviewers?<br><br>Was interrater agreement (κ statistic) assessed?<br><br>d. Risk of Bias Assessment<br>The manuscript states that JBI cross-sectional appraisal tools were used.<br><br>However:<br><br>The results of the appraisal should be summarized in the Results section.	transparency, reproducibility, and methodological rigor

A table (S1) is referenced but not fully interpreted or summarized in the text.<br><br>e. Meta-analysis Specifics<br>To ensure reproducibility, the authors must specify:<br><br>Thresholds for heterogeneity (I^2 cutoff)<br><br>Handling of zero-event studies<br><br>Whether proportions were transformed (logit, Freeman–Tukey double arcsine)<br><br>This is essential because meta-analysis of proportions is highly sensitive to transformation choice.<br><br>f. Ethics<br>Systematic reviews do not require IRB approval.<br>However, the authors should state this explicitly:<br><br>"As this study used published data only, institutional review board approval was not required."	
**3. Results**<br>Strengths<br><br>PRISMA flow diagram is clear and appropriate.<br><br>Characteristics of included studies are well organized.<br><br>Tables and figures illustrate study design, sample sources, and regional distribution.<br><br>Forest plots are visually clear and appropriately formatted.<br><br>Areas for Improvement<br><br>a. Chronological Alignment with Objectives<br>Results should be structured into clear subsections:<br><br>Description of included studies<br><br>Resistance patterns by antibiotic class<br><br>Resistance patterns by pathogen<br><br>Heterogeneity assessment<br><br>Currently, there is overlap between description and interpretation of findings.<br><br>b. Over-reporting raw proportions<br>Many values are repeated excessively in the narrative. For a meta-analysis, the text should highlight:<br><br>Most resistant pathogens	We thank the reviewer for the valuable comments on the Results section. The authors have revised the Results to improve chronological alignment with the study objectives, restructure the section into clear subsections, reduce repetition of raw proportions, clarify figure and table presentation, and define all abbreviations through appropriate footnotes

Most resistant antibiotic classes<br><br>Most consistent patterns across studies<br><br>Any observable trend over time<br><br>c. Figures and Tables<br><br>Some figure numbers overlap (Fig 1 appears referenced twice).<br><br>Figures S1–S14 are referenced but not described in adequate detail.<br><br>d. Missing Footnotes and Abbreviation Definitions<br>Table 1 uses acronyms (EMD, IPD, SSI, PROM) without footnotes. Standard reporting requires defining all abbreviations.	
**4. Discussion**<br><br>Strengths.<br><br>Adequately interprets overall findings.<br><br>Connects resistance patterns to clinical relevance and treatment guidelines.<br><br>Compares findings with regional and global literature.<br><br>Identifies implications for empirical prescribing guidelines.<br><br>Areas for Improvement.<br><br>a. Too Much Repetition of Results<br>Several paragraphs simply restate the same numeric values from the Results section.<br>The Discussion should instead emphasize:<br><br>Why resistance is high<br><br>Mechanisms (antibiotic misuse, OTC availability, weak stewardship, hygiene gaps)<br><br>Regional differences<br><br>Policy impact<br><br>b. Missing Interpretation of Heterogeneity<br>High heterogeneity (>75%) was identified in most analyses but not discussed.<br>The authors must explain:<br><br>Possible causes: variation in study designs, regions, testing methods	We thank the reviewer for the constructive comments on the Discussion section. The authors have revised the Discussion to reduce repetition of results, strengthen interpretation of the findings, explain the observed heterogeneity, and incorporate forward-looking recommendations aligned with Tanzania’s National Action Plan on Antimicrobial Resistance. In addition, limitations related to publication bias have been explicitly acknowledged

Implications for pooled estimates<br><br>c. Lack of Forward-Facing Recommendations<br>Given Tanzania has a National AMR Action Plan, the authors should recommend:<br><br>Strengthening surveillance systems<br><br>Restricting OTC antibiotic access<br><br>Enhancing stewardship in hospitals<br><br>Improving laboratory diagnostics<br><br>Updating STG/NEMLIT guidelines<br><br>d. Missing Consideration of Publication Bias<br>Even though publication bias tests for proportion meta-analysis are limited, authors should state:<br><br>Why funnel plot asymmetry was not tested<br><br>Limitations related to publication bias	
**5. Limitations**<br><br>This section is present but needs expansion to include:<br><br>High heterogeneity across studies<br><br>Overrepresentation of urban hospital data<br><br>Underrepresentation of rural facilities<br><br>Variation in laboratory testing standards<br><br>Possible publication bias<br><br>No time-trend analysis due to inconsistent reporting years	Thank you for the observation, the authors have revised the section
**6. Conclusion**<br>Strengths<br><br>Summarizes major findings.<br><br>Emphasizes importance of stewardship and surveillance.<br><br>Areas for Improvement<br><br>Should more clearly reflect the primary objective: pooled prevalence.<br><br>Should include concrete policy recommendations.<br><br>Should avoid adding new information not discussed earlier.	Thank you for the observation, the authors have revised the section

Should follow PRISMA standard: one paragraph summarizing findings, implications, and future needs.	
**SUMMARY FOR AUTHOR**<br><br>To improve the manuscript for resubmission, the authors should:<br><br>Expand the introduction with stronger literature review and clearer objectives.<br><br>Provide a more reproducible and transparent search strategy.<br><br>Clarify duplicate removal and data extraction steps.<br><br>Explicitly describe meta-analysis transformations and heterogeneity interpretation.<br><br>Structure the results more clearly and reduce redundancy.<br><br>Strengthen the discussion with causal explanations, regional differences, policy implications, and stewardship recommendations.<br><br>Add a complete limitations section.<br><br>Improve clarity and grammar throughout.<br><br>Provide complete data availability in supplemental files.<br><br>Ensure all figures, abbreviations, and footnotes meet reporting standards.<br><br>Ensure all figures are accessible or in the paper.	Thank you, this has been taken into account
