# Supplementary material for: Prevalence of antimicrobial resistance in Tanzania: A systematic review and meta-analysis
Source: PLoS One. 2026 Apr 21;21(4):e0346433. doi: 10.1371/journal.pone.0346433 (PMC13098951; doi:10.1371/journal.pone.0346433)
Supplement: Attachment — Submitted filename: Reviewed comments.docx [file pone.0346433.s005.docx]

Thank you for the opportunity to review this manuscript. Overall, it was well written. Below are issues I observed and recommendations provided.

**Abstract**

1. Please include justification for conducting the study in the abstract
2. Adjust the statement “Heterogeneity was assessed using forest plots”. A forest plot is not used to assess heterogeneity. Heterogeneity is assessed using I² statistic.
3. Is the prevalence of antimicrobial resistance defined by descriptive or meta-analysis?
4. Adjust the grammar in this statement: “*Resistance to meropenem was lowest among Escherichia coli (0.04 [0.01–0.10]) and Klebsiella spp. (0.07 [0.03–0.15]), with an overall pooled 37 resistance to the ESKAPE-E pathogen of (0.11[0.06–0.19])*.”
5. Specify who this recommendation is targeted at “*The variability in resistance patterns underscore the need to re-evaluate empirical treatment protocols (STG/NEMLIT) to ensure effective treatment regimens, strengthening antimicrobial stewardship, enhancing surveillance systems, and promote rational antibiotic use.”*

**Introduction**

6. **Line 47 – 59**: Can you quantify the burden of antimicrobial resistance globally and in Tanzania, respectively?
7. Clarify the language in this statement: “*This phenomenon is particularly noticeable in low- and middle-income countries, where antibiotic abuse is pervasive, infectious disease burdens are high, yet scarce healthcare resources*”
8. Provide references for the statement: “*A variety of factors contribute to the rising rates of Antimicrobial Resistance (AMR) in the country, including over-prescription of antibiotics, self-medication, and inadequate regulatory frameworks for antibiotic use.”*
9. *What do you mean by “urgency” in* ***line 68?***
10. Line 69 -70: describe and cite what other reviews have done or did not do. Also, cite some individual studies on antimicrobial resistance in Tanzania that you referred to in this section.
11. Address the grammar in this statement: *While several studies have reported resistance patterns for specific pathogens, therefore a consolidated analysis that captures the overall prevalence across the country was needed*

**Method**

12. I think the information on lines 91 – 98 should be part of your introduction
13. State what approach was used to define the research question. If you used the PICO approach, include it in your “Review procedures section.”
14. Justify why you focused only on studies across 10 years
15. In your selection criteria, is there a difference between *“studies reporting on the prevalence of antibiotic resistance in bacterial pathogens” and “studies focused on antibiotic resistance”*
16. Did you predefine the bacterial pathogens and antibiotics analyzed in this review? If yes, you should justify why they were selected
17. State how many reviewers conducted the quality assessment?
18. Include the statistical package you used to conduct descriptive statistics.
19. Usually, only the I² statistic is used to evaluate heterogeneity and not the forest plot.
20. What cut-off was used to determine the higher value for the I² statistic?

**Results**

21. More specific numbers should be used instead of percentages to report on the number of studies. When using percentages, maintain consistency in reporting. Also, ensure that the papers conducted at each location are cited.
22. **Line 186 – 190.** You mentioned 17 antibiotics but only listed 10. You should include the other seven. You should define the criteria that were used to categorize resistance as high or low.

**Discussion**

23. This is new; it was not mentioned in the result. Ensure that you discuss only the results presented.
24. I suggest you focus on interpreting the results rather than repeating them in the discussion section.
25. **Line 306: “**Reserved group**.”** This was not mentioned in the result**.** You should not bring up new terms or information in your discussion
26. You should also focus on connecting the findings of the study to the study objectives. The objectives seem lost somewhere in the discussion
27. You should provide explanations and reasons behind each finding from the study
28. “These findings are similar to the studies conducted in Iran, USA, India, and Africa (80 83), where higher resistance rates were reported, highlighting the potential regional differences in antibiotic use and resistance patterns. ” Please, provide more information on these studies.
29. You may also want to add some recommendations to the discussion

**Conclusion**

30. “Last resort” is this referring to the reserved group mentioned in the discussion section? If yes, ensure consistency in the terms used across the manuscript.

Thanks
