# Supplementary material for: Prevalence of antimicrobial resistance in Tanzania: A systematic review and meta-analysis
Source: PLoS One. 2026 Apr 21;21(4):e0346433. doi: 10.1371/journal.pone.0346433 (PMC13098951; doi:10.1371/journal.pone.0346433)
Supplement: Attachment — Submitted filename: Response to reviewers_PLOS one2.docx [file pone.0346433.s008.docx]

| Review of manuscript- PLOS One  **Tittle:** **Prevalence of Antimicrobial Resistance in Tanzania: A Systematic Review and Meta-Analysis** | |
| --- | --- |
| Dear Editor, The authors thank the editor for reviewing our work. Please see our point-by-point response to the reviewers’ comments below and find our revised manuscript attached. On behalf of the co-authors, Kind regards, Charles Basil Kafaiya *kafaiyacharles@gmail.com* *Research Scientist, National Institute for Medical Research, Dar es salaam, Tanzania* \| | |
| **Additional editors comments** | **Response** |
| 1. Since **AMR** was defined in line 57, please use the abbreviation **AMR** in place of antimicrobial resistance in subsequent occurrences (e.g., lines 79, 177, etc.). Please ensure that the use of this abbreviation is applied consistently throughout the manuscript 2. Line 184, add "." at the end of the sentence 3. Please remove the subheading **“Limitations.”** The study limitations should instead be incorporated into the final paragraph of the **Discussion** section 4. Please replace the subheading **“Conclusion, Recommendations, and Policy Implications”** with **“Conclusions.”** Any recommendations and policy implications should be integrated into the final paragraph of the **Conclusions** section 5. In line 361, please delete the abbreviation "(UTIs)" which was not used anywhere else in the manuscript and not listed under the list of abbreviations | We thank the reviewer for constructive comments and suggestions. This has helped to improve the clarity and consistency of the manuscript. All comments have been carefully addressed, and the recommended revisions have been incorporated in the revised manuscript |
| **Reviewer 2** | **Response** |
| The integration of a quality assessment tool would be best discussed after data synthesis, analysis and reporting section  Line 165-166 “Discrepancies between reviewers were resolved through discussion, and when consensus was not reached, a third reviewer adjudicated.” You can also state that the methodological quality of the included studies was independently assessed by two reviewers using the JBI Critical Appraisal Checklist for Prevalence Studies, and any discrepancies were resolved through consensus  Limitations: You mention heterogeneity is "likely due to differences in... antimicrobial susceptibility testing (AST) practices". You could specify if this refers to different guidelines, such as CLSI vs. EUCAST, as these are common sources of variation in AMR studies. Additionally, the phrase "formal assessment of publication bias was not performed due to methodological limitations" could be more specific. You might mention that standard tests (like Egger's test) are often underpowered or inappropriate for proportions/prevalence meta-analyses  Conclusion, recommendations, and policy implications: In the first sentence, there is a missing comma between "bloodstream infections" and "pneumonia”. Additionally, there is a typographical error in lines 367 and 368 | We thank the reviewer for the constructive comments and suggestions, we have incorporated the suggestions and resolved the comments accordingly |
| **Reviewer 3** | **Response** |
| The only minor issue I noticed was with the abbreviations. Since there is a section defining abbreviations, I suggest removing the definitions from the other sections to avoid repetition | Thank you for this observation. We have reviewed and addressed the issue accordingly |
