# Supplementary material for: Prevalence of antimicrobial resistance in Tanzania: A systematic review and meta-analysis
Source: PLoS One. 2026 Apr 21;21(4):e0346433. doi: 10.1371/journal.pone.0346433 (PMC13098951; doi:10.1371/journal.pone.0346433)
Supplement: Attachment — Submitted filename: Reviewer Comments.pdf [file pone.0346433.s007.pdf]

Reviewer Recommendation and Comments:

Manuscript Number PONE-D-25-41665R1

The integration of a quality assessment tool would be best discussed after data synthesis, analysis and reporting section.

Line 165-166 “Discrepancies between reviewers were resolved through discussion, and when consensus was not reached, a third reviewer adjudicated.” You can also state that the methodological quality of the included studies was independently assessed by two reviewers using the JBI Critical Appraisal Checklist for Prevalence Studies, and any discrepancies were resolved through consensus.

Limitations: You mention heterogeneity is "likely due to differences in... antimicrobial susceptibility testing (AST) practices". You could specify if this refers to different guidelines, such as CLSI vs. EUCAST, as these are common sources of variation in AMR studies. Additionally, The phrase "formal assessment of publication bias was not performed due to methodological limitations" could be more specific. You might mention that standard tests (like Egger's test) are often underpowered or inappropriate for proportions/prevalence meta-analyses.

Conclusion, recommendations, and policy implications: In the first sentence, there is a missing comma between "bloodstream infections" and "pneumonia”. Additionally, there is a typographical error in lines 367 and 368.
